# Benign subcutaneous emphysema: a rare and challenging entity *a case report and review of the literature*

**DOI:** 10.1080/23320885.2021.1984922

**Published:** 2021-10-01

**Authors:** Samuel M. Christen, Joerg G. Gruenert, Stefan Winsauer

**Affiliations:** Department of Hand, Plastic and Reconstructive Surgery, Kantonsspital St. Gallen, St. Gallen, Switzerland

**Keywords:** Benign subcutaneous emphysema, necrotizing soft tissue infection, gas gangrene, gas-forming bacteria

## Abstract

We report the case of a craftsman who developed a rapidly progressive subcutaneous emphysema of his forearm after a minor stab injury into the palm of his hand. Based on our case report we discuss differential diagnosis and management of acute subcutaneous emphysema.

## Introduction

Subcutaneous emphysema (SE) is defined by an accumulation of gas along fascial planes in the subcutis. The combination of an acute swelling with crepitus on physical examination and a subcutaneous gas collection on radiographs points to a necrotizing soft tissue infection (NSTI) [1]. With an incidence of 4 to 15.5 cases per 100,000 population, the NSTI is a rare but severe condition with very high morbidity and mortality [2,3]. While an NSTI is typically associated with an external trauma that inoculates a cutaneous or environmental germ into the deep soft tissue, some case reports of spontaneous NSTI are reported in the literature [4]. NSTI is characterized by a rapidly progressive course with extensive soft tissue destruction and systemic toxicity requiring immediate and aggressive medical and surgical treatment [5]. However, non-infectious etiologies for SE exist, like the presence of air in the soft tissue after a surgical procedure, a thoracic trauma or an accidental percutaneous injection of air (high-pressure injection injury) [6–9]. In addition, some case reports on an SE after a minor skin lesion can be found in the literature [10–16]. Diagnosis and management of these so-called benign subcutaneous emphysema (BSCE) can be challenging [17,18]. While the misdiagnosis of an NSTI in non-infectious SE may lead to an unnecessary and aggressive surgical treatment, the failure to identify a necrotizing infection usually results in limb amputation or even death.

## Case report

**Figure 1.**
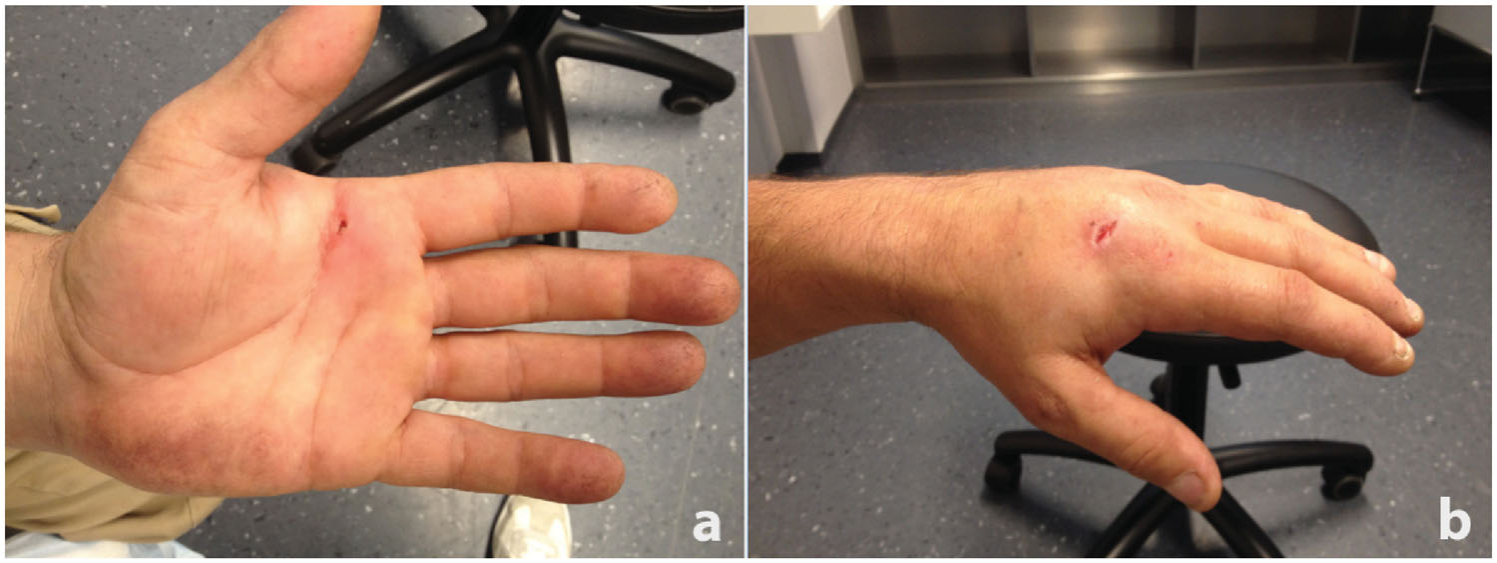
(a) Minor skin lesion with mild perifocal redness on the palm of the left hand. (b) Important swelling of the back of the hand (the visible skin lesion stems from a previous injury).

**Figure 2.**
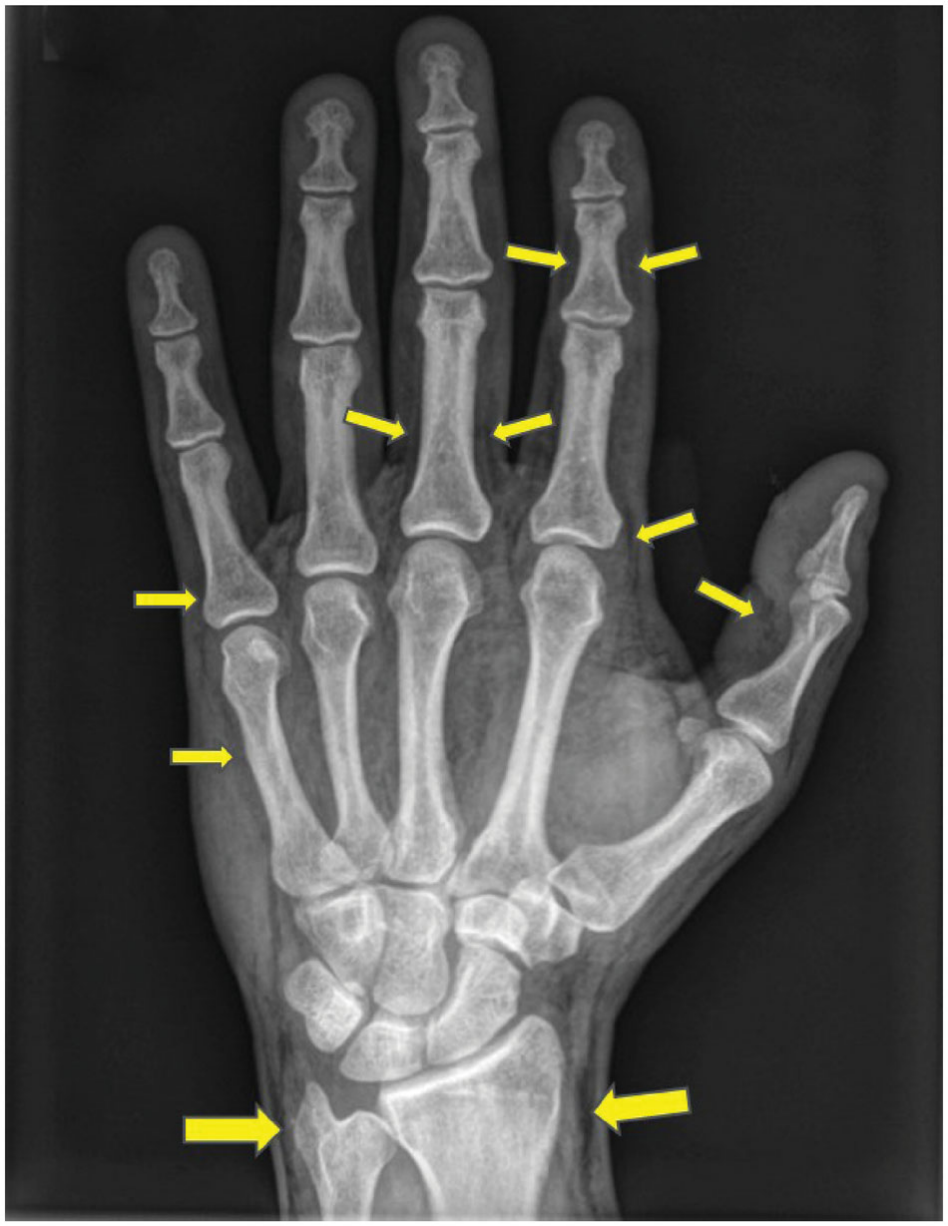
X-ray 6 h after trauma showing marked subcutaneous emphysema extending to the tip of the fingers and towards the forearm. The arrows depict the extension of the air along the epifascial planes.

A 56-year-old flooring installer sustained a wood splinter injury in his left palm while examining the processed wooden floor surface. He immediately removed the splinter (0.5 × 4 cm) himself. About 20 min later he noticed an increasing swelling with crackling sensations of the affected hand. Within minutes, this spread up to his forearm. The patient was admitted to our clinic 6 h after the injury in good general condition, vital signs were stable and no fever was present. On clinical examination, a distinct painless swelling from the hand to the distal forearm was present. Apart from mild perifocal redness and tenderness around the small penetration wound, there were no signs of infection and no skin changes (Figure 1). No pain was triggered by the passive and active motion of the finger or the wrist joint. A marked crackling could be palpated from the tips of the fingers to the elbow. The X-ray of the hand exhibited extensive subcutaneous emphysema (Figure 2). Apart from a slightly elevated C-reactive protein (13 mg/l), the blood samples were inconspicuous. According to the LRINEC score, the risk for the presence of necrotizing fasciitis was low (Table 1). Nevertheless, the patient received a high-dose i.v. antibiotic therapy with amoxicilline/clavulanic acid supplemented with clindamycin (3 × 900 mg/24h) as toxin-suppressor and was admitted immediately to the OR for surgical debridement. Another wood splinter of 2 cm length was detected, leading to the flexor tendons of the index finger, but no complex structures were harmed. No pus or suspicious fluid collection could be found. The surrounding soft tissue was vital and well perfused. Nevertheless, tissue samples were obtained for bacteriological analysis. The postoperative clinical course was uneventful, the emphysema diminished spontaneously and the patient was discharged 48 h after the injury. The wound swabs showed no bacterial growth.

**Table 1.** Laboratory Risk Indicator for Necrotizing Fasciitis (LRINEC) score.

Variable (units)	Score	Case report
C-reactive protein (mg/L)		
<150	0	13
≥150	4	
Total leucocyte count (thousands/mm^3^)		
<15	0	9.0
15 − 25	1	
>25	2	
Hemoglobin (g/dL)		
>13.5	0	14.8
11 − 13.5	1	
<13.5	2	
Serum sodium (mmol/L)		
≥135	0	136
<135	2	
Serum creatinine (μ mol/L)		
≤141	0	76
>141	2	
Glucose (mmol/L)		
≤10	0	5.3
>10	1	

The LRINEC score is an additional parameter that helps to distinguish benign emphysema from necrotizing fasciitis:

The values of our case (green) are shown in the right column of the table with a score of 0.

Total score of ≤5 = low risk, 6–7 = intermediate risk, ≥8 = high risk.

**Figure 3.**
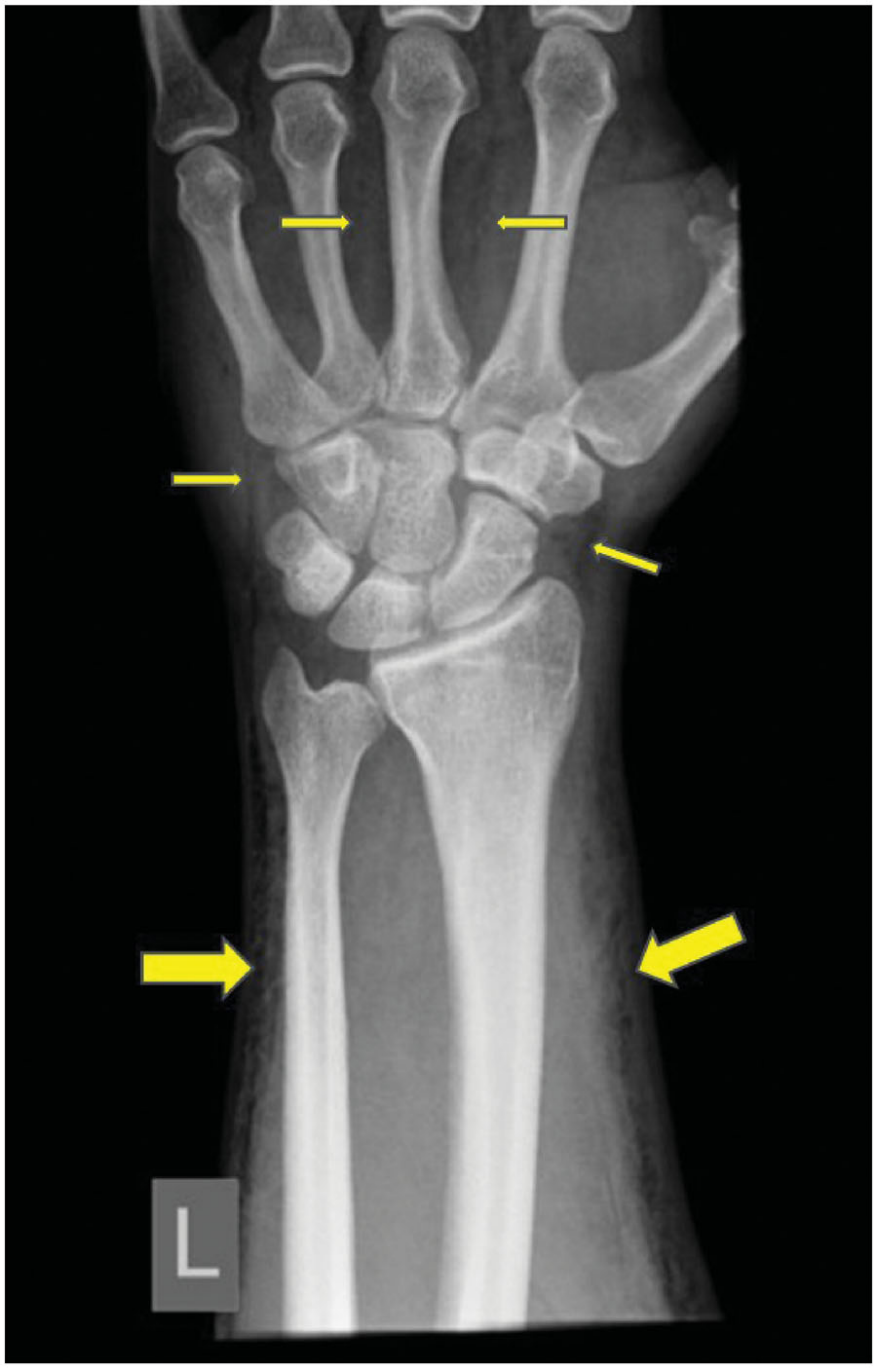
X-ray after the contusion of the left hand 7 years ago with visible subcutaneous air collections emphysema of the left arm.

Interestingly, the same patient had suffered from emphysema of the same arm 7 years ago after a contusion of the left wrist without visible skin lesions or a fracture (Figure 3). The patient had been treated in the medical department of our hospital at that time. He had been covered with antibiotics and had been discharged after a short period of observation. The past medical history is otherwise unremarkable. The patient has no chronic disease and is not on regular medication.

## Discussion

Only a few reports of non-infectious SE after minor skin lesions exist [10–16]. The non-infectious emphysema has to be clearly distinguished from potentially life-threatening infections with gas-forming germs such as clostridium perfringens, pyogenic streptococcus, coliform or anaerobic spore-forming bacteria [1,19]. The clostridial gas gangrene usually occurs with a delay of 12–18 h after the initial trauma and develops in a fulminant manner. Non-clostridial gas gangrene proceeds more insidiously over few days and mostly affects patients with an underlying chronic disease such as diabetes, chronic organ insufficiency or malignancies [19]. The most common form of an NSTI is monomicrobial necrotizing fasciitis with group A streptococcus (GAS) [1]. Often, a superficial skin lesion can be identified. The GAS infection begins with relatively mild local skin changes (discoloration, erythema and swelling); significant crepitus develops after 12–18 h. Over 24 h the affected skin becomes purple and bullae appear. Typically, patients suffer from severe pain (‘pain out of proportion’) and the inflammation spreads rapidly accompanied by progressive tissue destruction within a few hours. The patients deteriorate dramatically, showing systemic signs of toxicity (high fever, hemodynamic instability, organ failure). The clinical evaluation and decision-making can be supplemented by routinely performed blood samples. The LRINEC score (tab. 1) was introduced in 2004 and proved a helpful adjunct for risk stratification in such cases [20,21]. A score of more than 8 points is strongly predictive for an NSTI (PPV 93.4%). However, a low LRINEC score does not exclude necrotizing fasciitis. Therefore, the threshold for early surgical exploration should be low. In a clinically unstable patient supportive therapy and immediate treatment is vital including high-dose antibiotic therapy (with toxin-suppressor), exploration of the wound with aggressive soft-tissue debridement and eventually hyperbaric oxygen therapy [22,23]. Hu N et al. suggest a novel therapeutic strategy of vacuum therapy combined with continuous irrigation with potassium permanganate [24]. Despite advances in intensive care medicine and general awareness of NSTI, mortality rates remain high, even with appropriate treatment [25].

**Figure 4.**
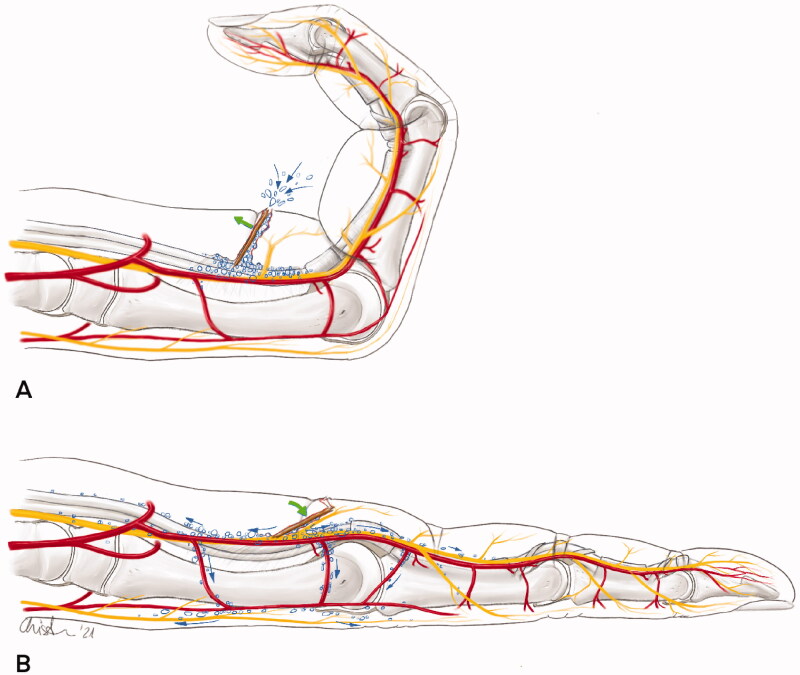
Illustration depicting the supposed mechanism for the rapidly spreading subcutaneous emphysema: (A) In flexion, the wound is held open by the wood splinter and air can enter. (B) When straightening it, the wound closes by a “trap door” phenomenon and the air is pushed forward along the neurovascular structures.

In BSCE the period between the initial trauma and the development of the swelling and crepitus is significantly shorter than in NSTI [8]. In contrast to infectious emphysema, the SE develops without any delay within a few minutes to hours. Mild local skin changes (erythema) and tenderness may be present as well. The pathophysiology of the BSCE is purely mechanical. The incorporation of air into the subcutaneous tissue may be explained by a one-way valve mechanism [26]. As a consequence of moving the affected limb, air can enter through the small skin wound and spreads out along those subcutaneous layers with the least resistance (e.g. neurovascular bundles). Some anatomical sites, like the first interdigital space, seem to be more prone to the development of a BSCE [27]. The differential diagnosis of non-infectious, physically induced SE remains wide. It can occur after trauma, surgical dissection, or accidental injection of air (high-pressure injection injury, mishap during intravenous infusion). In some cases of non-infectious SE, severe pain may be present due to the development of a compartment syndrome, particularly after a high-pressure injection injury [28]. Furthermore, a factitious manipulation related to the Munchhausen syndrome has to be kept in mind if the underlying cause is not apparent [29]. In our case, the SE was probably facilitated by the fact that the splinter formed a connection from the surface to the flexor tendon and the neurovascular bundles along the foreign body (Figure 4).

The reason for the spontaneous emphysema without any obvious skin lesion from which our patient had suffered 7 years ago remains unclear. No other comparable case can be found in the current literature. An individual predisposition for subcutaneous emphysema can be discussed but is purely speculative.

## Conclusion

At an early stage, the distinction between an infectious SE and a BSCE can be difficult. We, therefore, recommend admission of patients with signs of a BSCE for close monitoring and re-evaluation. If in doubt, initiation of a broad-spectrum antibiotic therapy and immediate surgical exploration is imperative. Further imaging (e.g. MRI) should not delay surgery. Usually, limited local exploration of the initial wound or the most prominent swelling is sufficient to rule out an NSTI. Typical intraoperative findings of necrotizing fasciitis would be friability of the fascia, blunt dissection with minimal resistance (‘finger test’) and presence of grey-coloured fluid (‘dishwater fluid’) [1,17]. If none of these findings is present, local irrigation and drainage are sufficient.
